# Primary neurons lacking the SNAREs vti1a and vti1b show altered neuronal development

**DOI:** 10.1186/s13064-022-00168-2

**Published:** 2022-11-22

**Authors:** Christian Bollmann, Susanne Schöning, Katharina Kotschnew, Julia Grosse, Nicole Heitzig, Gabriele Fischer von Mollard

**Affiliations:** Biochemistry III, Department of Chemistry, Bielefeld University, Bielefeld, Germany

**Keywords:** Neurite outgrowth, Golgi outpost, Postsynaptic density, Enlargeosome, Y27632, SNARE, vti1a, vti1b

## Abstract

**Background:**

Neurons are highly specialized cells with a complex morphology generated by various membrane trafficking steps. They contain Golgi outposts in dendrites, which are formed from somatic Golgi tubules. In trafficking membrane fusion is mediated by a specific combination of SNARE proteins. A functional SNARE complex contains four different helices, one from each SNARE subfamily (R-, Qa, Qb and Qc). Loss of the two Qb SNAREs vti1a and vti1b from the Golgi apparatus and endosomes leads to death at birth in mice with massive neurodegeneration in peripheral ganglia and defective axon tracts.

**Methods:**

Hippocampal and cortical neurons were isolated from *Vti1a*^*−/−*^ *Vti1b*^*−/−*^ double deficient, *Vti1a*^*−/−*^ *Vti1b*^*+/−*^, *Vti1a*^*+/−*^ *Vti1b*^*−/−*^ and *Vti1a*^*+/−*^ *Vti1b*^*+/−*^ double heterozygous embryos. Neurite outgrowth was determined in cortical neurons and after stimulation with several neurotrophic factors or the Rho-associated protein kinase ROCK inhibitor Y27632, which induces exocytosis of enlargeosomes, in hippocampal neurons. Moreover, postsynaptic densities were isolated from embryonic *Vti1a*^*−/−*^ *Vti1b*^*−/−*^ and *Vti1a*^*+/−*^ *Vti1b*^*+/−*^ control forebrains and analyzed by western blotting.

**Results:**

Golgi outposts were present in *Vti1a*^*−/−*^ *Vti1b*^*+/−*^ and *Vti1a*^*+/−*^ *Vti1b*^*−/−*^ dendrites of hippocampal neurons but not detected in the absence of vti1a and vti1b. The length of neurites was significantly shorter in double deficient cortical neurons. These defects were not observed in *Vti1a*^*−/−*^ *Vti1b*^*+/−*^ and *Vti1a*^*+/−*^ *Vti1b*^*−/−*^ neurons. NGF, BDNF, NT-3, GDNF or Y27632 as stimulator of enlargeosome secretion did not increase the neurite length in double deficient hippocampal neurons. *Vti1a*^*−/−*^ *Vti1b*^*−/−*^ postsynaptic densities contained similar amounts of scaffold proteins, AMPA receptors and NMDA receptors compared to *Vti1a*^*+/−*^ *Vti1b*^*+/−*^, but much more TrkB, which is the receptor for BDNF.

**Conclusion:**

The absence of Golgi outposts did not affect the amount of AMPA and NMDA receptors in postsynaptic densities. Even though TrkB was enriched, BDNF was not able to stimulate neurite elongation in *Vti1a*^*−/−*^ *Vti1b*^*−/−*^ neurons. Vti1a or vti1b function as the missing Qb-SNARE together with VAMP-4 (R-SNARE), syntaxin 16 (Qa-SNARE) and syntaxin 6 (Qc-SNARE) in induced neurite outgrowth. Our data show the importance of vti1a or vti1b for two pathways of neurite elongation.

**Supplementary Information:**

The online version contains supplementary material available at 10.1186/s13064-022-00168-2.

## Background

Neuronal development requires complex membrane trafficking events, which are regulated by members of conserved protein families. Specific combinations of SNARE proteins (soluble N-ethylmaleimide-sensitive-factor attachment receptor) mediate membrane fusion [1]. These SNARE proteins assemble into four helix bundles containing different SNARE motifs belonging to the four different subfamilies defined by sequence similarities (R, Qa, Qb and Qc-SNARE). Certain SNARE proteins such as SNAP-23 and SNAP-25 contain a Qb and a Qc-SNARE motif and require only a Qa and a R-SNARE for complex formation. We are interested in the neuronal function of the ubiquitously expressed Qb-SNAREs vti1a and vti1b [2, 3]. Vti1a is localized to the Golgi apparatus, the trans Golgi network (TGN), early endosomes and synaptic vesicles [4]. It forms a SNARE complex with VAMP-4 (R-SNARE), syntaxin 6 (Qc) and syntaxin 16 or syntaxin 13 (Qa) functioning in early endosome fusion and retrograde transport to the TGN. Vti1a deficient mice display defects in dense-core vesicle biogenesis in adrenal chromaffin cells [5]. This defect is not aggravated by additional deletion of *Vti1b* nor is it observed in vti1b deficient mice. Vti1b is found predominantly in late endosomes and lysosomes but also co-localizes with vti1a in the Golgi area. The SNARE complex partners of vti1b are VAMP-7 (also called Ti-VAMP) or VAMP-8 (R-SNAREs), syntaxin 8 (Qc) and syntaxin 7 (Qa) [6, 7]. These complexes have a role in fusion with the lysosome or late endosome, respectively. Exocytosis of lytic granules is impaired in cytotoxic T cells from *Vti1b*^*−/−*^ mice [8]. While *Vti1a*^*−/−*^ and *Vti1b*^*−/−*^ mice are viable *Vti1a*^*−/−*^ *Vti1b*^*−/−*^ double deficient mice die at birth suggesting a partially redundant function for vti1a and vti1b [9, 10]. These double knockout (DKO) embryos show massive impairments in neuronal development. Neurons are reduced in several ganglia, major axon tracts are missing and cortical layer 5 is malformed [10, 11]. Neurite outgrowth is reduced in cultured DKO hippocampal neurons [10]. Different pathways requiring different SNAREs contribute to neurite outgrowth. The SNARE complex mediating regulated exocytosis consisting of synaptobrevin 2 (also called VAMP-2), syntaxin 1 and SNAP-25 is also involved in neurite outgrowth [12]. Interference with VAMP-7/Ti-VAMP or SNAP-23 reduces neurite length [13]. Incorporation of enlargeosomes, a special membrane compartment, results in very rapid elongation of neurites. The SNAREs VAMP-4, syntaxin 6 and SNAP-23 have been implicated in this pathway [14]. Neurotrophic factors can stimulate growth of neurites as shown with BDNF, NGF, NT3 and GDNF for hippocampal neurons in cell culture [15–17]. Interference with VAMP-4 and syntaxin 6 reduces NGF-stimulated neurite outgrowth in the neuroendocrine PC12 cell line [18, 19]. NGF, BDNF and NT3 bind to their receptors TrkA, TrkB and TrkC, respectively, which can signal via endosomes [20]. In the absence of vti1a and vti1b cultured neurons display reduced exit of synaptic vesicle proteins and dense core-vesicle cargo from the Golgi, which reduces regulated secretion of these vesicles [21]. It was observed that the Golgi structure is altered and its area reduced in cultured hippocampal neurons as well as in hippocampal sections [21]. Therefore, vti1a and vti1b-associated intracellular trafficking events appear to play a crucial role in successful neuronal maturation. It needs to be further analyzed which trafficking steps are influenced.

Here we investigated whether neurite outgrowth can be stimulated by neurotrophic factors and by activation of the enlargeosome incorporation. As BDNF did not stimulate neurite elongation in *Vti1a*^*−/−*^ *Vti1b*^*−/−*^ neurons, we studied the amounts of TrkB receptor in post-synaptic densities isolated from brain extracts. We also compared AMPA receptor and NMDA receptor because AMPAR is delivered via the somatic Golgi, NMDAR via Golgi outposts [22].

## Methods

### Animals

Generation and first characterization of mouse strains have been previously described: *Vti1b*^*−/−*^ [9], *Vti1a*^*−/−*^ and *Vti1a*^*−/−*^ *Vti1b*^*−/−*^ [10]. Animals were bred into the C57BL/6 mouse background and embryos were prepared on embryonic developmental day 15.5 (E15.5) or 18.5 (E18.5) after timed matings (*Vti1a*^*−/−*^ *Vti1b*^*+/−*^ or *Vti1*^*+/−*^ *Vti1b*^*−/−*^ females with *Vti1a*^*+/−*^ *Vti1b*^*−/−*^ or *Vti1a*^*−/−*^ *Vti1b*^*+/−*^ males, respectively).

### Culture of cortical and hippocampal neurons

Cortical or hippocampal regions were isolated at embryonic development day E15.5 for cortical neurons and NT-3 stimulation or E18.5 for all other experiments. The neuronal regions were trypsinized (Trypsin-EDTA, Lonza) after extraction, washed in Neurobasal or Neurobasal A media (Gibco) supplemented with either 2% B27 (Gibco) or 2% N2 (Gibco) as well as 1% Glutamax (Gibco) and 0.2% penicillin/streptomycin (PAA) [23]. Neurons were further separated by pipetting through narrowed Pasteur pipettes. They were placed on poly-L-lysine coated culture dishes and further cultured at 37 °C and 5% CO_2_.

### Immunocytochemistry

**Fig. 1:**
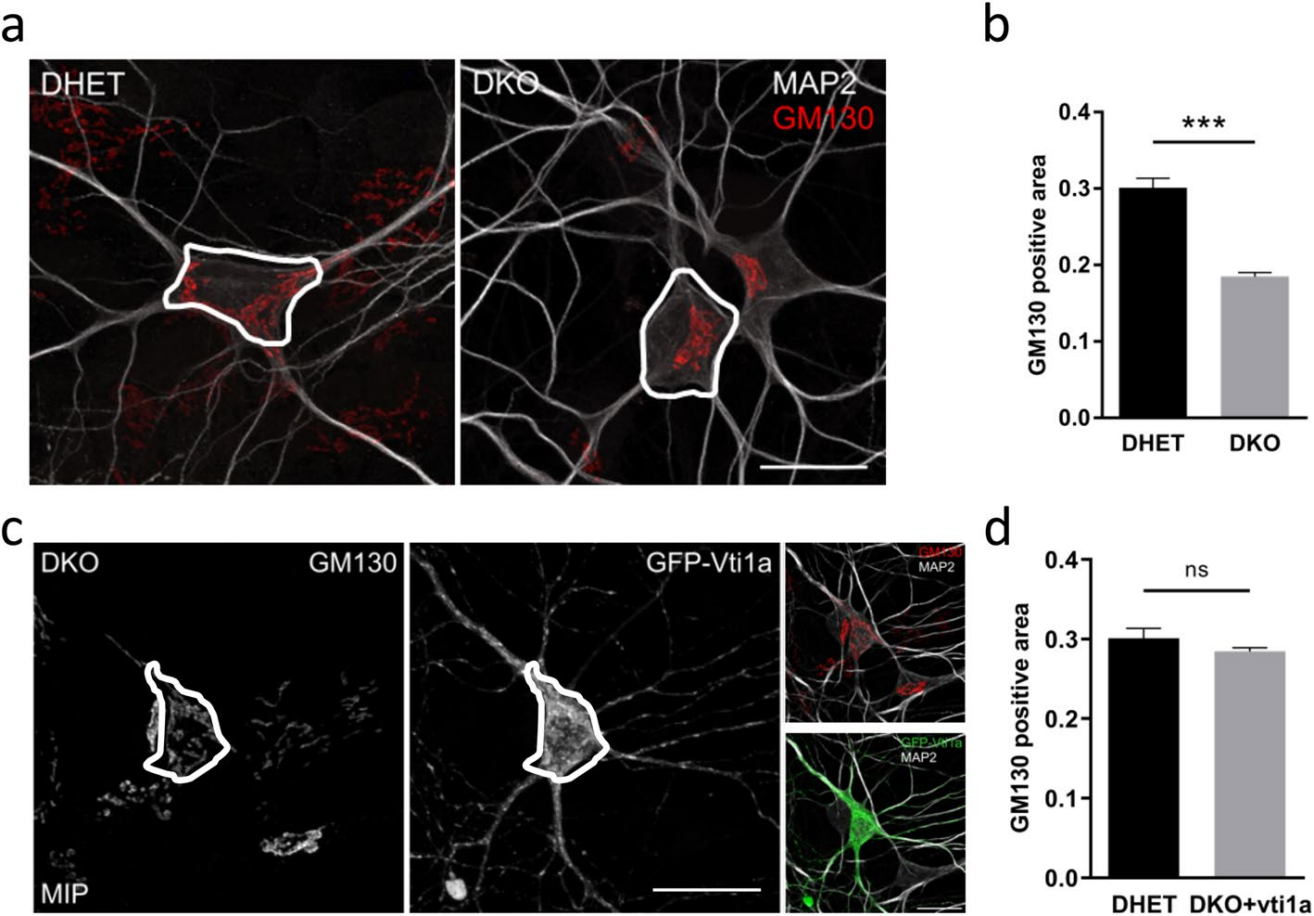
Deviating somatic distribution of Golgi in DKO neurons. Hippocampal neurons were isolated at E18.5 and cultivated for 8 days in vitro (8 DIV). (a) DHET and DKO neurons were identified with anti-MAP2 antibodies (white) and the Golgi apparatus were labeled with anti-GM130 antibodies (red). The white line was added in ImageJ to mark the cell body. Confocal Z-stacks were taken and maximum intensity projections (MIP) were presented. (b) Stacks of pictures were taken and the pictures with the largest Golgi area were quantified (GM130 pixels above threshold within the cell body divided by area of the cell body). (c) DKO neurons were transfected with GFP-Vti1a at 3 DIV and were further cultivated for 5 DIV. Cells were stained and (d) analyzed as described above. *N* = 3 embryos with > 15 cells per experiment ± SEM ***: *P* < 0.001, *P* > 0.05 not significant (ns) unpaired student’s t-test, scale bars (a) and (c) 20 μm

Hippocampal neurons were cultured on glass coverslips precoated with 0.1 mg/mL poly-L-lysine (Sigma-Aldrich). Cells were fixed in 4% paraformaldehyde in PBS or in 4% paraformaldehyde + 4% sucrose in PBS and permeabilized with 0.1% or 0.02% Triton X-100 in PBS. Unspecific antibody binding sites were blocked with blocking solution (1% goat-sera in PBS). Primary antibody incubation was overnight at 4 °C or 3 hours at room temperature with the following antibodies: βIII-Tubulin (1:1000, G7121-Promega), GM130 (1:400, 610822-BD Transduction Laboratories), Golgin-97 (1:60, D8P2K-CellSignaling), PDI (1:400, 1D3-Enzo Life Science) and MAP2 (1:1000, ab5392-Abcam). Cells were washed and incubated with fluorochrome-conjugated secondary antibodies (1:400, Dianova) for 1 h. Cells were washed, and the nuclei stained with Hoechst 33342 (Invitrogen) diluted 1:1000 in PBS. Coverslips were washed and mounted with Mowiol 4–88 (Sigma-Aldrich) onto slides. Images were taken with a fluorescence microscope (Leica, IM1000) and further Z-stacks were obtained on a confocal microscope (Zeiss, LSM700). The image with the largest GM130-positive area in a Z-stack was opened in ImageJ for quantification of the Golgi area. The cell body including the broad base of neurites was encircled to separate between soma and dendrites (examples in Fig. 1). Pixels with GM130 signal above background were determined in this region and divided by the total number of pixels in this region.

### Stimulation with neurotrophic factors

For NT-3 stimulation hippocampal neurons (E15.5) were prepared and cultured in Neurobasal N2 media (Gibco) for 1 h. For stimulation cells were covered with Neurobasal A with added N2 supplement (Gibco) and 30 ng/mL NT-3 (N1905, recombinant, human, Sigma Aldrich) for 48 h. Then cells were fixed and immunostained for ßIII-tubulin. For the stimulation of hippocampal neurons with BDNF, GDNF and NGF (E18.5) embryos were prepared as described above. Cells were cultured in sera free media for 24 h before adding 100 ng/mL NGF (450–01, recombinant, human, Preprotech), 25 ng/mL BDNF (B3795, recombinant, human, Sigma Aldrich) or 50 ng/mL GDNF (G1777, recombinant, human, Sigma Aldrich and 450–10, Preprotech) for 24 h. Afterwards cells were fixed and immunostained for ßIII-tubulin. As a control for the stimulation cells were cultivated in media without the addition of neurotrophic factors. The length of the longest neurite of each neuron was determined in *ImageJ* and the average length of the longest neurite for at least 100 neurons for each embryo was calculated.

### ROCK-inhibition in hippocampal neurons through Y27632

Hippocampal neurons (E18.5) were prepared as described and cultured for 15 h. The media was changed to Neurobasal media containing 1% FCS and 60 μM Y27632. The stimulation lasted for 3 h and afterwards cells were fixed with PFA and immunostained for ßIII-tubulin. Controls were cultivated in Neurobasal B27 media containing 1% FCS but no Y27632.

### Calcium phosphate transfection

Primary hippocampal DKO neurons were cultivated and transfected using a modified calcium phosphate coprecipitation method [24] at DIV3 with a GFP-Vti1a plasmid based on pEGFP-C1 (pFvM141). Preconditioned Neurobasal supplemented media was stored, and the media of the cells was changed to Neurobasal media without additives. For two transfections 2 μg of DNA and 3.5–5 μL sterile CaCl_2_-Solution (2,5 M) were added to 50 μL 2x BES-Solution (280 mM NaCl, 1.5 mM Na_2_HPO_4_ and 50 mM N,N-Bis(2-hydroxyethyl)-2-aminoethanesulfonic acid pH 7.1). The solution was topped up to 100 μL with sterile ddH_2_O, incubated for 20–25 min and added to 900 μL Neurobasal media. Mixtures were given to the neurons and these were incubated at 37 °C and 5% CO_2_ for 60–90 min. Cells were washed with Hank’s Balanced Salt Solution (HBSS, BioWest) and cultivated for 1 h in Neurobasal media. Cells were washed again before they were further cultivated in their original media for 5 days until they were fixed.

### Isolation of postsynaptic densities

Forebrain regions from E18.5 embryos were extracted and frozen. Postsynaptic densities were isolated according to [25]. DKO forebrains yielded less protein in the homogenate and lower amounts of postsynaptic density proteins compared to DHET. Briefly, 8 DHET or 11 DKO forebrains were pooled and mechanically homogenized in osmotic buffer solution A (4 mM HEPES pH 7.4, 0.32 M sucrose, 1 mM MgCl_2_ and 0.5 mM CaCl_2_). Postnuclear supernatant (PNS) was obtained after a centrifugation step (1.400x g) from homogenized tissues. The crude synaptosomal fraction (P2) was the pellet fraction after a centrifugation step (13.800x g). The pellet was resuspended in solution B (4 mM HEPES pH 7.4 with 0.32 M sucrose), which was further separated through ultracentrifugation (43.000x g) using a discontinuous sucrose gradient containing most of P2 and 3 mL of each 0.85 M, 1.0 M and 1.2 M sucrose solutions. Afterwards the postsynaptic density containing layer was obtained between 1.0 M and 1.2 M sucrose and 0.5% (v/v) Triton X-100 was added. After a centrifugation step (43.000x g) the pellet contained the PSD, which was further analyzed by immunoblotting.

### SDS-PAGE and immunoblotting

SDS-PAGE was performed according to the method of Laemmli [26] using an 8% SDS-Gel. Proteins were electrophoretically transferred to nitrocellulose membranes by wet blot according to the method described by Towbin [27]. Membranes were blocked in TBST blocking solution (150 mM NaCl, 25 mM Tris-HCl pH 7.4 and 0.1% Tween) with 5% BSA (Roth) followed by primary antibody incubation with one of the following antibodies: actin (Gonsior et al. 1999), Shank3 (D5K6R-CellSignaling), SAP102 (A7R8L-CellSignaling), PSD95 (7E3-CellSignaling), NR2B (D8E10-CellSignaling), TrkB (80E3-CellSignaling) or GluR1 (ab31232-Abcam) diluted 1:1000. Proteins were quantified with the SuperSignal® kit (Thermo Scientific) and densitometry analysis was performed with ImageJ (National Institute of Health, Bethesda).

### Statistics

Statistical significance was calculated using Prism7 (GraphPad) and data sets were analyzed by student’s t-test, or ANOVA as appropriate.

## Results

### Vti1a/Vti1b deficient neurons exhibit altered Golgi positioning in soma

Our previous data showed a reduced GM130-positive area in CA1 hippocampal regions of E18.5 DKO compared to *Vti1a*^*+/−*^ *Vti1b*^*+/−*^ controls (double heterozygote, DHET) [21]. This reduced Golgi area was also observed in DKO hippocampal neurons at 5 or 14 days in vitro (DIV5 or DIV14) [21]. Here we isolated E18.5 hippocampal neurons and cultivated them for 8 or 12 days in vitro (DIV8 or DIV12) for a further analysis of the Golgi and a first investigation of the ER. Neurons were labeled with anti-GM130 antibodies. Figure 1a indicated a more scattered GM130-positive signal in DHET neurons compared to the DKO, where it was located more closely to the nucleus. Measuring the GM130-positive areas in the soma of the hippocampal cells validated this impression and represented a significantly reduced GM130-stained region in the absence of vti1a and vti1b (Fig. 1b). The Golgi area looked unaffected in *Vti1a*^*−/−*^ *Vti1*^*+/−*^ and *Vti1a*^*+/−*^ *Vti1b*^*−/−*^ hippocampal neurons (Additional file 1: Fig. A1) demonstrating that a single intact allele of *Vti1a* or *Vti1b* was sufficient for correct Golgi morphology.

We next wanted to assess if we can rescue this effect by a vti1a-overexpression in DKO neurons. Indeed, successfully transfected GFP-vti1a-positive neurons showed a widespread GM130-signal within the soma, which was comparable to the Golgi area in DHET neurons (Fig. 1c,d). The subcellular distribution of GFP-vti1a was similar to that of vti1a in wild-type neurons.

Neurons were co-stained with antibodies against GM130 and Golgin-97 at DIV8 to compare Golgi and TGN morphology (Additional file 1: Fig. A2a, A2b). The area stained for Golgin-97 was also smaller in DKO than in DHET neurons indicating that the TGN was also affected. GM130 and Golgin-97 were in close proximity in DHET as well as in DKO neurons. These data indicate that the spatial arrangement between Golgi and TGN was intact in DKO neurons.

Taken together, the SNAREs vti1a and vti1b seemed to play a role in somatic GM130-positive Golgi distribution and localization.

### Golgi outpost formation is disturbed in DKO neurons

**Fig. 2:**
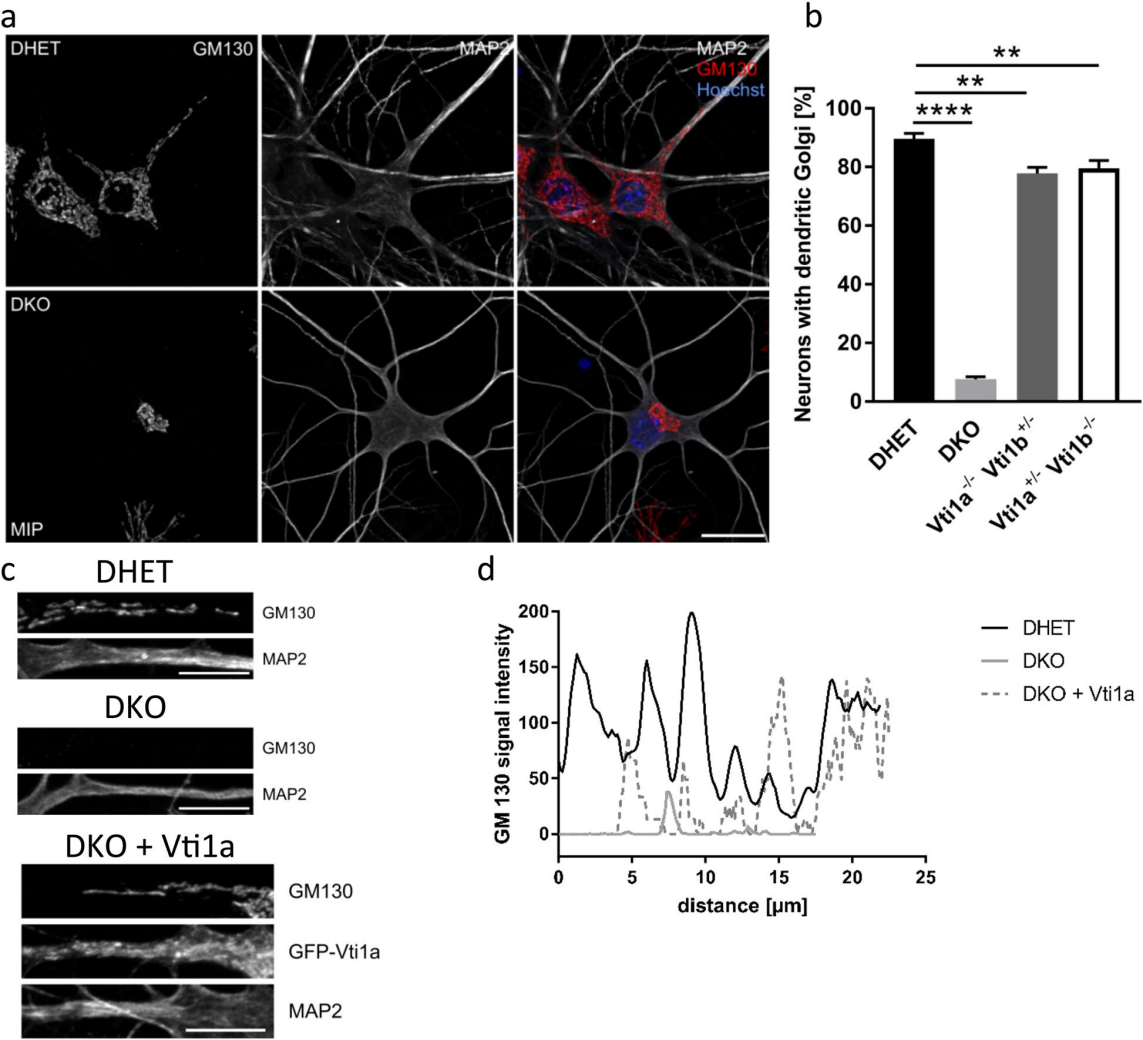
DKO dendrites lack Golgi outposts. Hippocampal neurons were isolated at E18.5 and cultivated for 12 days in vitro (12 DIV). (a) DHET and DKO neurons were double-stained with primary antibodies against GM130 (red) and MAP2 (white). The nuclei were marked with Hoechst (blue). Confocal Z-stacks were taken and maximum intensity projections (MIP) were presented. (b) The percentage of cells with Golgi extensions into dendrites was determined for DHET and DKO as well as for *Vti1a*^*−/−*^ *Vti1b*^*+/−*^ and *Vti1a*^*+/ -*^ *Vti1b*^*−/−*^ at DIV12. N = 3–5 mean of at least 100 cells per experiment. Bars are mean of the mean of 4 different DHET, 5 DKO, 3 *Vti1a*^*−/−*^ *Vti1b*^*+/−*^ and 4 *Vti1a*^*+/ -*^ *Vti1b*^*−/−*^ embryos ± SEM. **: *P* < 0.01, ****: *P* < 0.0001 one-way ANOVA. (c) DKO neurons were transfected with GFP-Vti1a at 3 DIV and were further cultivated for 5 DIV. Cells were stained together with DHET and DKO (DIV8) as described above and dendrites were magnified. (d) A representative line scan through one dendrite per condition of GM130 signal is plotted over the length of the dendrite. Scale bars (a) 20 μm and (c) 10 μm

Based on our results, we wondered whether vti1a vti1b double-deficient neurons are still able to form Golgi outposts, which are essential for dendritic development and preservation [28, 29]. Tubules extend from the somatic Golgi into the major dendrite and are cleaved off to form Golgi outpost during maturation of hippocampal neurons in culture [30]. Therefore, hippocampal neurons at DIV12 were analyzed for GM130 staining that extended into dendrites. In DHET neurons GM130-positive organelles were clearly visible in MAP2-marked dendrites (Fig. 2a). In contrast, almost no GM130 signal could be detected in DKO dendrites. Nearly 90% of DHET neurons exhibited Golgi extensions or outposts, whereas less than 10% of DKO neurons were able to form these dendritic organelles (Fig. 2b). *Vti1a*^*−/−*^ *Vti1b*^*+/−*^ and *Vti1a*^*+/−*^ *Vti1b*^*−/−*^ neurons contained less Golgi extensions or outposts compared to DHET, but still almost 80% showed GM130-positive regions in dendrites (Additional file 1: Fig. A1, Fig. 2b). Dendritic Golgi was already detected in 80% of DHET but only in 20% of DKO neurons at DIV8 (Additional file 1: Fig. A3). The fraction of neurons with Golgi extensions was slightly reduced in *Vti1a*^*+/−*^ *Vti1b*^*−/−*^ but not altered in *Vti1a*^*−/−*^ *Vti1*^*+/−*^ compared to DHET neurons.

Again, DKO neurons were transiently transfected with GFP-Vti1a and cells were cultivated for 8 days. Magnifications of the dendrites revealed GM130-positive areas in case of vti1a overexpression in the DKO dendrites (Fig. 2 c). Areas of high signal intensity were observed in these as well as in DHET cells in line scans throughout the dendrites (Fig. 2d).

Because ER signals detected with antibodies directed against the ER marker PDI could be observed in DHET and DKO dendrites, the ER seemed unaffected by the loss of both SNARE proteins (Additional file 1: Fig. A4a – A4d).

In summary, the two SNAREs vti1a and vti1b appeared to be involved in Golgi outpost formation and seemed to be able to replace each other. However, absence of vti1a and vti1b did not affect the ER distribution within dendrites.

### Neurite outgrowth was reduced in *Vti1a*^*−/−*^*Vti1b*^*−/−*^ cortical neurons

**Fig. 3:**
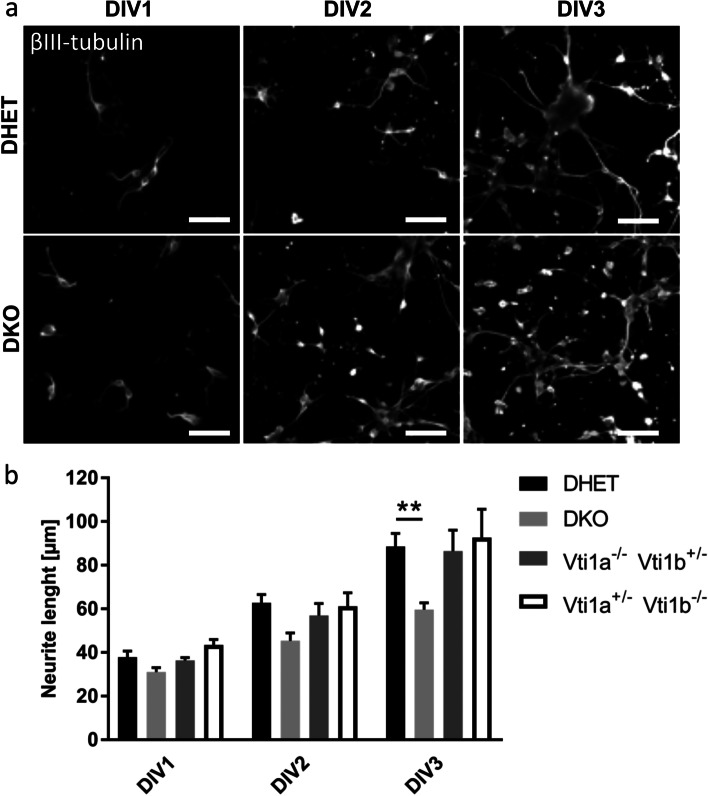
Neurite outgrowth was decreased in DKO cortical neurons. Cortical neurons were cultivated from E15.5 embryos on poly L-lysine for one, two or three days (DIV1, DIV2 or DIV3). (a) DHET and DKO neurons were stained with an antibody directed against βIII-tubulin (white). The scale bar represents 30 μm. (b) To investigate the neurite outgrowth the length of the longest neurite of each neuron was determined in *ImageJ* and the average length of the longest neurite for at least 100 neurons for each embryo was calculated. Neurons of DHET, DKO, *Vti1a*^*−/−*^ *Vti1b*^*+/−*^ and *Vti1a*^*+/ -*^ *Vti1b*^*−/−*^ were analyzed. At least 100 cells were measured from the following number of embryos: DHET DIV1 *n* = 4, DIV2 *n* = 12, DIV3 *n* = 11; DKO DIV1 to DIV3 each n = 4; *Vti1a*^*−/−*^ *Vti1b*^*+/−*^ DIV1 *n* = 6 DIV2 and DIV3 *n* = 9; *Vti1a*^*+/ -*^ *Vti1b*^*−/−*^ DIV1 *n* = 2 DIV2 and DIV3 *n* = 6. Bars represents the mean ± SEM, **: *p* < 0.01, two-way ANOVA

Because Golgi and Golgi outposts are important for dendritic development [31] and cortical neurons contain Golgi outposts [32] we wondered about neurite outgrowth in cortical neurons [10]. Therefore, cortical neurons were isolated from E15.5 DKO, DHET, *Vti1a*^*−/−*^ *Vti1*^*+/−*^ and *Vti1a*^*+/−*^ *Vti1b*^*−/−*^ embryos. Neurons were cultivated on poly L-lysine for one, two or three days (DIV1, DIV2 or DIV3), fixed and identified by immunofluorescence staining for ßIII-tubulin (Fig. 3a). During cultivation the cortical neurons showed a neurite outgrowth and started to build a neuronal network. On DIV1 and 2 the neurite length seemed to be comparable between DHET and DKO in microscopic pictures. On DIV3 a difference between both genotypes appeared. Apparently, the DKO neurons had shorter neurites compared to DHET. The length of the longest neurite was determined (Fig. 3b). On DIV1 the average neurite length was similar between these genotypes. On DIV2 the DHET neurites tended to be longer with 63 ± 4 μm compared to the DKO (45 ± 4 μm). Neurites of DKO neurons (60 ± 3 μm) were significantly shorter than neurites of DHET cells (89 ± 6 μm) after DIV3. The *Vti1a*^*−/−*^ *Vti1b*^*+/−*^ and *Vti1a*^*+/−*^ *Vti1b*^*−/−*^ neurons behaved like the DHET with an average length of 86 ± 10 μm and 93 ± 13 μm, respectively. Similar results were obtained after cultivation on laminin (data not shown).

Summarized, DKO neurons were capable to form neurites but they already showed a reduced neurite growth after three days in vitro.

### The enlargeosome pathway of neurite outgrowth was affected in *Vti1a*^*−/−*^*Vti1b*^*−/−*^ neurons

**Fig. 4:**
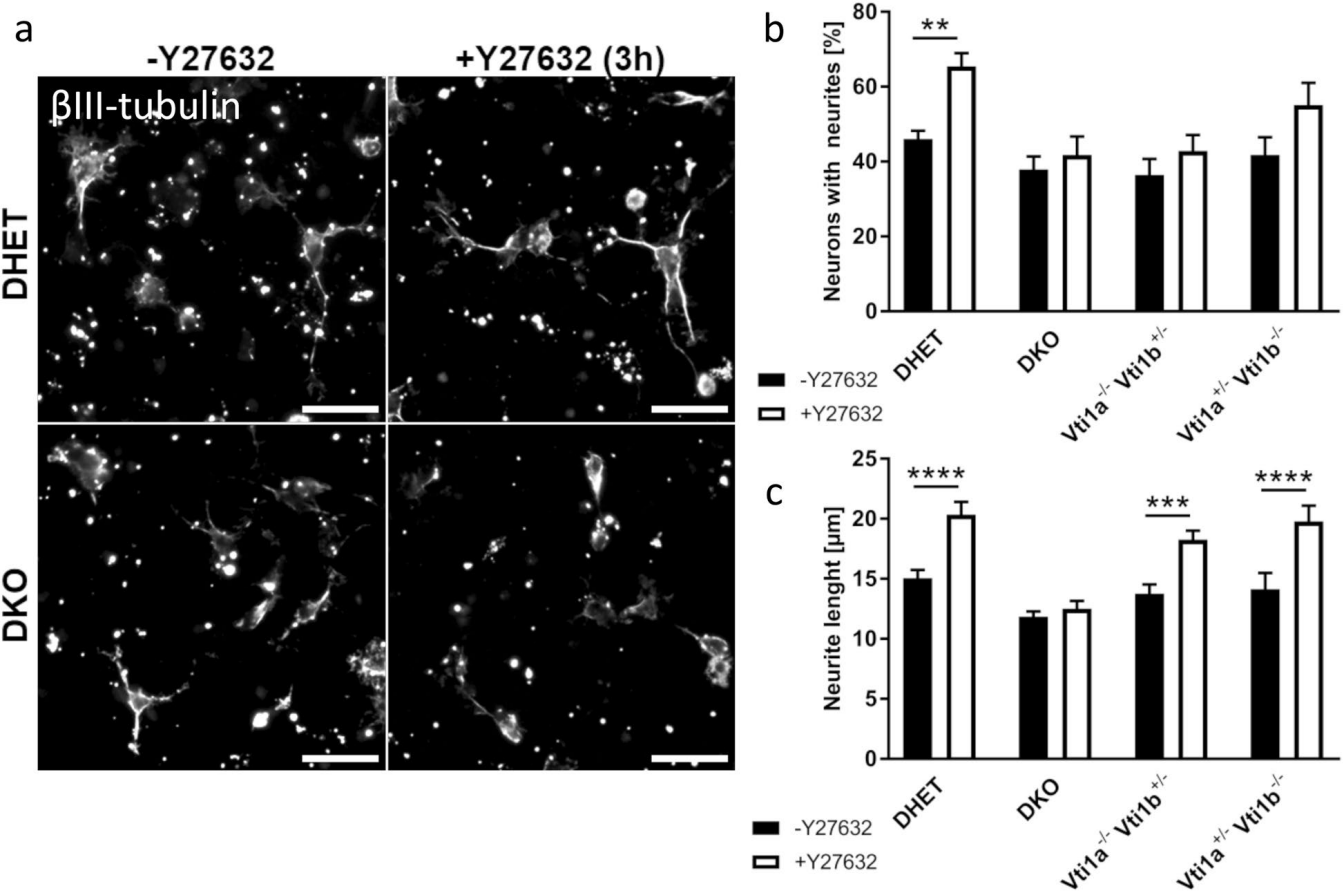
Enlargeosome mediated neurite outgrowth was reduced in DKO hippocampal neurons. Hippocampal neurons were isolated from E18.5 DHET, DKO, *Vti1a*^*−/−*^ *Vti1b*^*+/−*^ and *Vti1a*^*+/ -*^ *Vti1b*^*−/−*^ embryos and cultivated for 15 h followed by an incubation with or without 60 μM Y27632 for 3 h. (a) Y27632-treated and non-treated cells were stained for βIII-tubulin (white). Scale bar: 25 μM (b) The percentage of neurons with neurites after treatment was determined. (c) Neurite outgrowth was quantified by measuring the longest neurite per neuron and by calculation the average length for each embryo. At least 100 cells per genotype and treatment were analyzed (9 DHET, 6 DKO, 8 *Vti1a*^*−/−*^ *Vti1b*^*+/−*^ and 7 *Vti1a*^*+/ -*^ *Vti1b*^*−/−*^ embryos). Bars are the mean ± SEM, **: p < 0.01, ***: *p* < 0.001, ****: *p* < 0.0001, two-way ANOVA

Different pathways contribute to neurite outgrowth. One of those pathways is enlargeosome exocytosis which requires VAMP-4 and syntaxin 6 [14]. The enlargeosome-mediated neurite growth was examined because VAMP-4 and syntaxin 6 form a SNARE complex with vti1a [4]. Enlargeosome exocytosis and rapid neurite outgrowth can be induced by the drug Y27632, which is an inhibitor of the Rho-associated protein kinase (ROCK) [18]. To analyze the enlargeosome-mediated neurite growth E18.5 hippocampal neurons were cultivated for 15 h before incubation with or without 60 μM Y27632 for 3 h (Fig. 4). Untreated neurons had few or no neurites regardless of the presence of vti1a and vti1b. After treatment a neurite outgrowth could be recognized. In microscopic images DHET seemed to possess longer neurites after incubation with Y27632 than DKO cells. Quantification revealed that on the one hand, the inhibitor increased the fraction of neurons with neurites in DHET but not in DKO neurons (Fig. 4b). On the other hand, Y27632 increased the average length of the longest neurite of DHET cells (Fig. 4c). By contrast, DKO neurite elongation was not stimulated by Y27632 treatment. Surprisingly, *Vti1a*^*−/−*^ *Vti1b*^*+/−*^ and *Vti1a*^*+/−*^ *Vti1b*^*−/−*^ neurons did not react to the treatment with Y27632 with an increased fraction of neurons with neurites suggesting that lower levels of vti1a or vti1b provided by a single allele were not sufficient to initiate formation of neurites efficiently. Nevertheless, the *Vti1a*^*−/−*^ *Vti1b*^*+/−*^ and *Vti1a*^*+/−*^ *Vti1b*^*−/−*^ neurons with neurites show induced elongations comparable to that of DHET neurons.

These data indicate that the loss of vti1a and vti1b impaired the enlargeosome pathway.

### Neurotrophic factors did not stimulate neurite outgrowth in *Vti1a*^*−/−*^*Vti1b*^*−/−*^ neurons

**Fig. 5:**
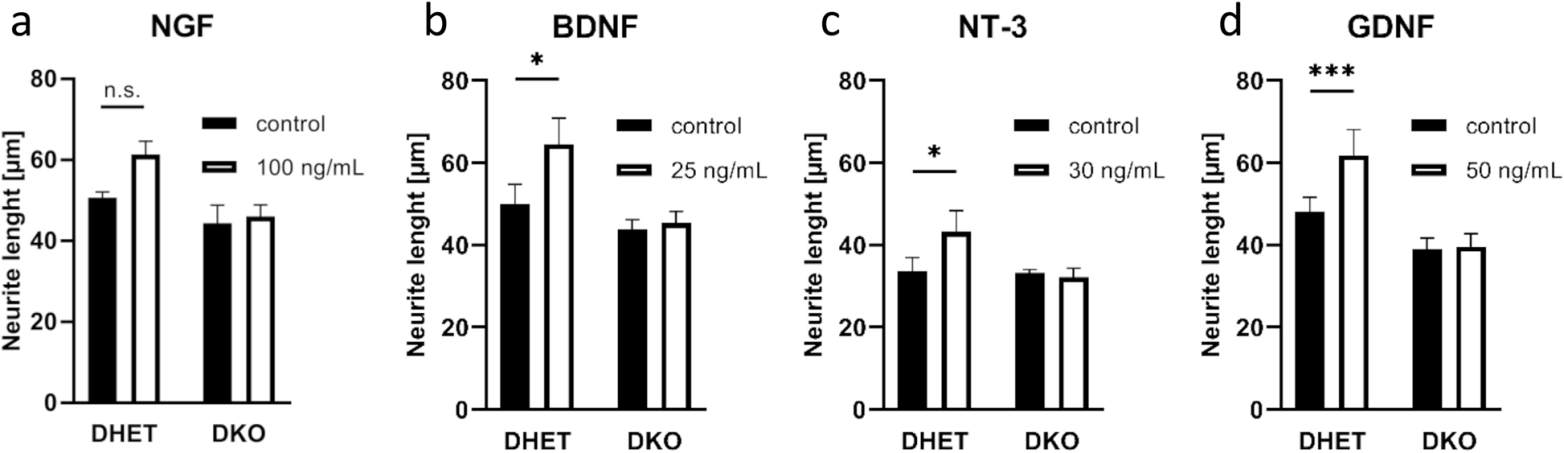
Neurotrophic factors did not induce neurite growth in DKO. Hippocampal neurons were isolated from E15.5 and E18.5 DHET or DKO embryos, cultivated for 24 h and treated with 100 ng/mL NGF (E18.5, a), 25 ng/mL BDNF (E18.5, b), or 50 ng/mL GDNF (E18.5, d) for 24 h or treated directly after preparation with 30 ng/mL NT-3 (E15.5, c) for 48 h. Neurite outgrowth was quantified by measuring the longest neurite per neuron and by calculation the average length for each embryo. At least 100 cells were measured per time point. Number of embryos: NGF: 3 DHET, 3 DKO; BDNF: 7 DHET, 9 DKO; NT-3: 3 DHET, 4 DKO; GDNF: 6 DHET, 6 DKO. The bars represent the mean ± SEM, *: *p* < 0.05, ***: p < 0.001 two-way ANOVA

Neurotrophic factors can stimulate neurite outgrowth in many neurons [15–17, 20, 33]. This was also shown for hippocampal neurons with BDNF, NGF, NT3 and GDNF [15–17, 20, 33]. As we reported before, neurite outgrowth is less pronounced in DKO than in DHET dorsal root ganglion explants after stimulation with the neurotrophin NGF [10]. We tested whether various neurotrophins (NGF, BDNF, NT-3) were able to stimulate neurite outgrowth in E15.5 or E18.5 DKO hippocampal neurons. As expected from previous studies, neurite length increased significantly upon treatment with the neurotrophins BDNF and NT-3 in DHET neurons (Fig. 5b, c) and slightly, but in these experiments not significantly with NGF (Fig. 5a). By contrast, DKO neurites did not respond to any of these factors. These results were independent of the embryonic development of the neurons (neurons isolated at E18.5 for NGF and BDNF, E15.5 for NT-3).

Moreover, members of the GDNF family of neurotrophic factors can also stimulate neurons [16]. Treating neurons with 50 ng/mL GDNF (Fig. 5d) induced longer neurites in DHET hippocampal neurons but was without effect in DKO cells, as well.

### Increased amounts of TrkB within DKO postsynaptic densities

As shown here, DKO neurons exhibited shorter neurites and did not react significantly to neurotrophic factors. The missing response to neurotrophic factors may be due to absence or mislocalization of their receptors. The BDNF receptor TrkB is localized to axons and dendrites of cultured hippocampal neurons as well as to postsynaptic densities (PSD) isolated from brains [34]. Hence, we have analyzed the protein composition within postsynaptic densities (PSD). DKO and DHET forebrains were homogenized and a postnuclear fraction (PNS) generated.

**Fig. 6:**
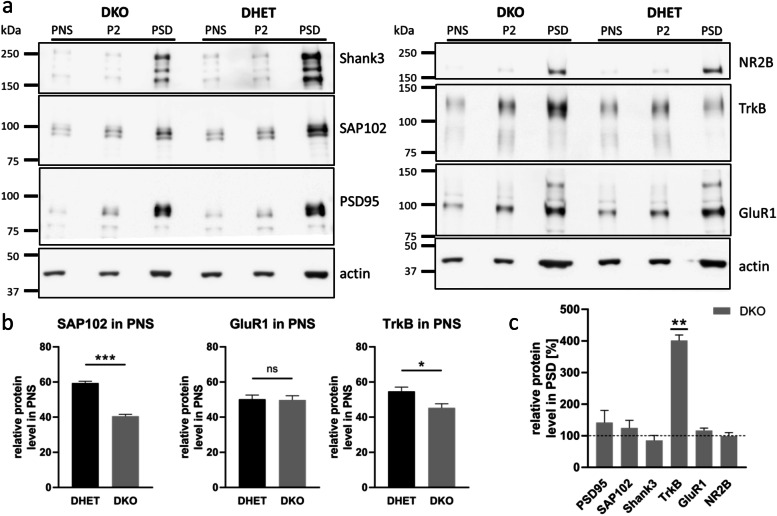
TrkB enrichment in the postsynaptic densities of DKO forebrain. Postsynaptic densities (PSD) were prepared from forebrains of E18.5 embryos. (a) Postnuclear supernatant (PNS), P2 (crude synaptosomes) and PSD fractions of DHET and DKO were analyzed by Western Blot analysis for Shank3, SAP102, PSD95, NR2B, TrkB and GluR1. Actin was used as loading control. Representative Western Blots were presented. (b) Ratios of SAP102, GluR1 and TrkB per actin were quantified for the PNS. *N* = 3 (SAP102 and TrkB) or 5 (GluR1), mean ± SD, ***: *P* < 0.001, *: *P* < 0.05, *P* > 0.05 not significant (ns) unpaired student’s t-test. (c) Ratio of PSD95, SAP102, Shank3 TrkB, GluR1 and NR2B per actin were quantified for PSD fraction. DHET were set to 100%. N = 3 (SAP102, TrkB and NR2B), 4 (PSD95) or 5 (GluR1 and Shank3), mean ± SD, **: *P* < 0.01 one-sample t-test

In Western Blot analysis the postsynaptic density scaffold protein SAP102 seemed to be reduced in the PNS fraction of DKO compared to DHET (Fig. 6a). Indeed, quantification of the SAP102 protein level demonstrated a significant reduction of the postsynaptic protein (Fig. 6b). AMPA receptor subunit GluR1 levels were not affected in the PNS, whereas TrkB amount was significantly reduced in the DKO forebrain compared to the DHET (Fig. 6b). Signals for the PSD scaffold proteins PSD95 and Shank3 as well as the NMDA receptor subunit NR2B were too low for quantification in all PNS fractions. The amounts of the endosomal SNAREs syntaxin 8, syntaxin 6 and VAMP-4 appeared to be unaffected by the absence of vti1a and vti1b in E18.5 brain lysates (Additional file 1: Fig. A5).

As expected, both crude synaptosomal P2 fractions showed increased signals for PSD95, TrkB or GluR1 compared to the PNS fraction (Fig. 6a). This revealed a successful enrichment of the nerve terminal membranes.

In the PSD fraction an obvious increase in signal intensity compared to PNS and P2 of all analyzed markers could be seen in DHET and DKO samples (Fig. 6a). Surprisingly, TrkB showed a greater enrichment in DKO than in DHET PSD fractions. Quantification demonstrated a significant 4-fold increase in the TrkB level in the absence of vti1a and vti1b (Fig. 6c). The enrichment of all other studied proteins was independent of the genotype.

Taken together, DKO neurons in the forebrain contained less SAP102 and TrkB. However, available TrkB in the double knockout brains was highly enriched within the region of the postsynaptic density.

## Discussion

In this work, we analyzed the impact of the two SNARE proteins vti1a and vti1b on neuronal differentiation in primary neurons from E15.5 and E18.5 embryos.

It is well established that pyramidal cells account for the vast majority of neurons in hippocampal cultures and they have a characteristic morphology distinct from interneurons. Neurons in DHET, DKO, *Vti1a*^*−/−*^ *Vti1b*^*+/−*^ and *Vti1a*^*+/−*^ *Vti1b*^*−/−*^ hippocampal cultures look similar suggesting that pyramidal cells were analyzed in all genotypes. DKO neurons display a more compact Golgi. Here we observed that the ER was not affected because the marker PDI was detectable in the dendrites independently of vti1a and vti1b. By contrast, Golgi extensions and outposts marked by GM-130 and TGN stained with Golgin-97 were not detected in *Vti1a*^*−/−*^ *Vti1b*^*−/−*^ dendrites. Only slightly less dendritic Golgi structures were observed in the absence of either vti1a or vti1b compared to DHET neurons suggesting a redundancy of vti1a and vti1b in this function. Golgi outposts in inhibitory neurons have been described only in Purkinje cells in the cerebellum during postnatal maturation [29]. As DKO embryos die at birth, these cells cannot be studied. Vti1a and vti1b are expressed in every tissue and cell type analyzed including chromaffine cells and several neuroblastoma cell lines. Therefore, it can be assumed that they are also present in inhibitory neurons. Vti1b mRNA has been detected in the cell bodies of Purkinje cells in adult mice by in situ hybridization [35]. Golgi outposts are formed from tubules derived from the somatic Golgi apparatus and tubule fission [30]. The perinuclear Golgi was more compact in DKO compared with DHET neurons in culture and in the hippocampus. This suggests direct or indirect problems with exit from the Golgi in DKO neurons including formation of Golgi tubules. Indirect effects seem more likely, because retrograde transport of cholera toxin from the plasma membrane via endosomes to the TGN was affected in DKO neurons in culture [21]. NMDA receptors are transported via Golgi outposts, while AMPA receptors reach the postsynaptic sites via the somatic Golgi [22]. Therefore, the amount of both receptors was analyzed in postsynaptic densities isolated from DKO and DHET forebrains. The subunits NR2B (NMDA receptor) and GluR1 (AMPA receptor) were detected in similar amounts in postsynaptic densities independent of the genotype. These data indicate that NMDA receptors are transported via an alternative route in the absence of vti1a and vti1b. Such an alternative secretory pathway in dendrites has been described before [36]. Transmembrane cargo such as GluA1 or neuroligin 1 can be transported from ER exit sites in dendrites to recycling endosomes before incorporation into the plasma membrane.

TrkB and the postsynaptic density scaffold protein SAP102 were less abundant in DKO forebrain postnuclear supernatants while PSD95 was too low for quantification. TrkB and the postsynaptic density scaffold protein PSD95 accumulate in the Golgi and are reduced in neurites of cultured DKO hippocampal neurons [21]. This trafficking defect may slightly destabilize these proteins resulting in the reduced levels detected in brain extracts. Surprisingly, TrkB was much more abundant in DKO compared to DHET postsynaptic densities while PSD95, SAP102 and Shank3 amounts were similar in the presence and absence of vti1a and vti1b. These differences are due to the different experimental approaches. Immunofluorescence of permeabilized cultured neurons reveals all proteins in the interior as well as on the cell surface. For isolation of postsynaptic densities, the forebrain is mechanically sheared rupturing cell bodies and neurites. Pre- and postsynaptic membranes reseal forming synaptosomes, which are pelleted as P2 due to their size. Dense membranes are isolated by sucrose gradient centrifugation and the Triton X-100 resistant pellets are the postsynaptic densities. TrkB amounts were similar in DHET and DKO P2 fractions. To account for the stronger enrichment in the last step TrkB must be much less abundant in DKO P2 membranes outside postsynaptic densities, for example in endosomes. Biogenesis of dense core vesicles is impaired in the absence of vti1a and vti1b resulting in reduced secretion of the neuropeptide Y in cultured neurons [21]. As BDNF is a cargo protein of dense core vesicles [37] there should be decreased secretion of BDNF in DKO brains. BDNF binding induces endocytosis of TrkB receptors [20]. Lower BDNF-induced endocytosis due to less BDNF could be the explanation for the elevated amounts of TrkB in DKO postsynaptic densities. An exposure to exogenous BDNF cannot be tested in this experimental setup because the postsynaptic densities are isolated from frozen embryonic brain. Due to limited material we did not test for phosphorylated pTrkB, TrkA or TrkC.

BDNF stimulated neurite elongation in DHET cultured hippocampal neurons but was without effect in DKO cells. This is surprising in context of a reduction of the TrkB amount by only a third in neurites of DKO cultured hippocampal neurons compared to DHET [21] as well as of elevated levels of TrkB in DKO brain postsynaptic densities. These data indicate that steps downstream of ligand binding to the receptor are affected in DKO neurons. Trk receptors are internalized and transported into signaling endosomes, which contain the protein network required for signaling [20]. BDNF stimulates dendritic growth only if clathrin mediated endocytosis is functional in hippocampal neurons at DIV 4 [38]. BDNF increases the co-localization of TrkB with rab5 and rab11 positive endosomes, influences endosome dynamics in hippocampal neurons and expression of dominant negative rab5 or rab11 reduce dendritic branching in hippocampal neurons at DIV 9 [39, 40]. These data demonstrate the importance of endosomes for BDNF stimulated dendritic growth. Endosomes may be defective in the absence of vti1a and vti1b explaining observed defects. NGF, NT-3 and GDNF also stimulated neurite elongation in DHET but not DKO hippocampal neurons. These defects were observed in hippocampal neurons isolated at embryonic day E18.5 (BDNF, NGF, GDNF) as well as E15.5 (NT-3) indicating that these defects are not due to problems in the late embryogenesis potentially impacting the response of the DKO neurons. It has been reported before that hippocampal neurites are enlongated by exposure to BDNF, NGF, NT-3 or GDNF in vitro [15–17]. Overexpression of a dominant negative domain of syntaxin 6 inhibited NGF-stimulated neurite growth in the neuroendocrine PC12 cells [19]. siRNA mediated silencing of VAMP-4 also reduced NGF-stimulated neurite elongation in PC12 cells [18]. As the R-SNARE VAMP-4 and the Qc-SNARE syntaxin 6 are SNARE partners of the Qb-SNARE vti1a these data indicate that these three proteins act together in neurotrophin stimulated neurite elongation. Syntaxin 16 probably is the Qa-SNARE of this complex since it is a partner of vti1a and neurite outgrowth in cortical neurons is reduced upon siRNA silencing of syntaxin 16 [41].

Enlargeosome mediated neurite outgrowth induced by the Rho-associated protein kinase ROCK inhibitor Y27632 was also defective in DKO hippocampal neurons. Interference with VAMP-4 or syntaxin 6 reduces enlargeosome exocytosis in PC12 cells [14]. Again, vti1a could provide the third SNARE helix to this complex. Vti1b was able to substitute for vti1a as Y27632 induced elongation of neurites in *Vti1a*^*−/−*^ *Vti1b*^*+/−*^ neurons. However, the number of cells with neurites was not increased in *Vti1a*^*−/−*^ *Vti1b*^*+/−*^ or *Vti1a*^*+/−*^ *Vti1b*^*−/−*^ neurons in contrast to DHET controls after 3 hours treatment with Y27632 indicating that one intact copy of either *Vti1a* or *Vti1b* may not produce sufficient protein for this fast reaction. *Vti1a*^*−/−*^ *Vti1b*^*+/−*^ or *Vti1a*^*+/−*^ *Vti1b*^*−/−*^ mice are fully viable and indistinguishable from DHET mice suggesting that longer time or other pathways for induction of neurites can compensate. Vti1a and vti1b may replace each other by binding to each others SNARE complex partners. Alternatively, the complete SNARE complex with four different SNAREs may fulfill missing functions. Interference with SNAP-23 also reduced enlargeosome mediated neurite outgrowth [14]. However, SNAP-23 as Qb-Qc-SNARE has to function in a SNARE complex distinct from a vti1a (Qb) and syntaxin 6 (Qc) complex.

### Conclusion

Taken together our data underline that vti1a or vti1b were required for several steps in neuronal development leading to the complex phenotype observed in DKO brains. Even a single functional allele of *Vti1a* or *Vti1b* was sufficient to prevent these phenotypes. Golgi outposts in dendrites did not form in DKO neurons, but three receptors still reached the postsynaptic densities indicating that alternative transport pathways were used. The BDNF receptor TrkB was much more abundant in postsynaptic densities isolated from brain. The inability of neurotrophic factors to stimulate neurite outgrowth in the presence of the BDNF receptor TrkB suggested defects in endocytosis, endosomes and signaling in the absence of vti1a and vti1b. Neurite outgrowth by enlargeosome exocytosis was not induced. VAMP-4 (R-SNARE), syntaxin 16 (Qa-SNARE) and synatxin 6 (Qc-SNARE) have been implicated in induced neurite outgrowth before. Our data indicate that their well established SNARE partner vti1a functioned as the missing Qb-SNARE but could be replaced by vti1b even though it is part of a different SNARE complex in the presence of vti1a or that the vti1b SNARE complex compensates for the defective function.

## Supplementary Information

**Additional file 1: Fig. A1.** Golgi structures in *Vti1a*^*−/−*^ *Vti1b*^*+/−*^ and *Vti1a*^*+/ -*^ *Vti1b*^*−/−*^ neurons are similar to DHET controls; **Fig. A2.** Altered TGN morphology in DKO neurons; **Fig. A3.** Less DKO neurons with Golgi extensions at DIV8; **Fig. A4.** The distribution of ER in DKO appeared to be unaffected. **Fig. A5.** The amounts of three endosomal SNAREs appeared to be unaffected by the absence of vti1a or vti1b in E18.5 brains.

## Data Availability

The datasets generated and analyzed during the current study are available from the corresponding author on reasonable request.

## Abbreviations

AMPAR: α-amino-3-hydroxy-5-methyl-4-isoxazolepropionic acid receptor
BDNF: brain-derived neurotrophic factor
DHET: *Vti1a* ^*+/−*^ *Vti1b*^*+/−*^ double-heterozygote
DIV: days in vitro
DKO: *Vti1a* ^*−/−*^ *Vti1b*^*−/−*^ double-knockout
FCS: fetal calf serum
GDNF: Glial cell line-derived neurotrophic factor
GluR1: Glutamate receptor 1, subunit of AMPAR
GM130: Golgi matrix protein 130
GO: Golgi outposts
MAP2: microtubuli associated protein
NGF: nerve growth factor
NMDA: N-methyl-D-aspartate receptor
NR2B: N-methyl D-aspartate receptor subtype 2B
NT-3: Neurotrophin − 3
P2: Synaptosomal fraction
PBS: Phosphate-buffered saline
PNS: Postnuclear supernatant
PSD: Postsynaptic density
PSD95: postsynaptic density protein 95
ROCK: Rho-associated coiled-coil forming protein serine/threonine kinase
SDS-PAGE: Sodium dodecylsulfate polyacrylamide gel electrophoresis
SEM: Standard error of the mean
Shank3: SH3 and multiple ankyrin repeat domains 3
SNARE: Soluble N-ethylmaleimide-sensitive-factor-attachment receptor
TGN: Trans Golgi network
TrkB: Tyrosine receptor kinase B
