# Supplementary material for: Primary neurons lacking the SNAREs vti1a and vti1b show altered neuronal development
Source: Neural Dev. 2022 Nov 22;17:12. doi: 10.1186/s13064-022-00168-2 (PMC9682837; doi:10.1186/s13064-022-00168-2)
Supplement: Supplementary file 1 — Additional file 1: Fig. A1. Golgi structures in Vti1a−/− Vti1b+/− and Vti1a+/ - Vti1b−/− neurons are similar to DHET controls; Fig. A2. Altered TGN morphology in DKO neurons; Fig. A3. Less DKO neurons with Golgi extensions at DIV8; Fig. A4. The distribution of ER in DKO appeared to be unaffected. Fig. A5. The amounts of three endosomal SNAREs appeared to be unaffected by the absence of vti1a or vti1b in E18.5 brains. [file 13064_2022_168_MOESM1_ESM.docx]

**
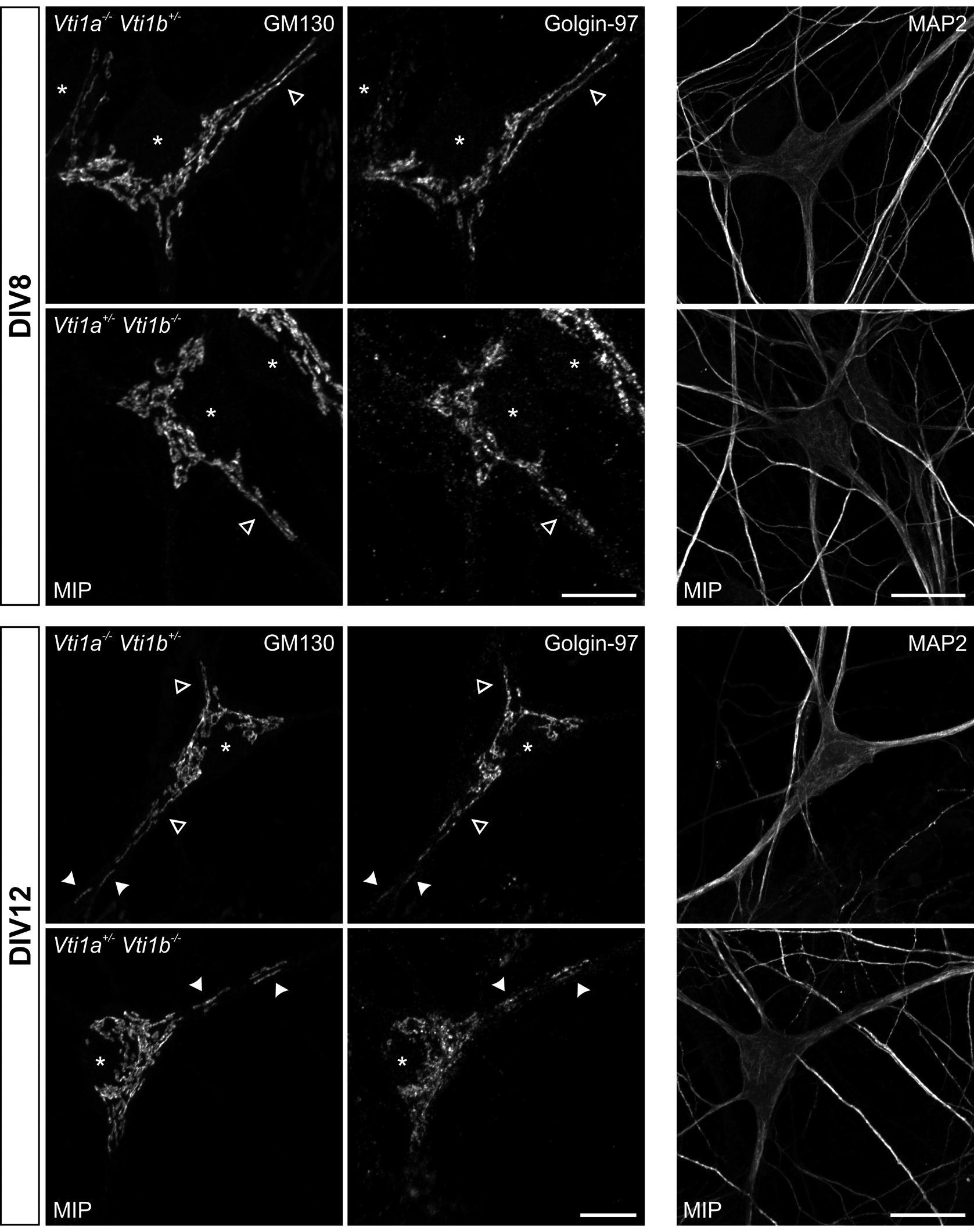
**

**Additional Figure A1: Golgi structures in *Vti1a^-/-^ Vti1b^+/-^* and *Vti1a^+/ ‑^ Vti1b^-/-^* neurons are similar to DHET controls.** Hippocampal neurons were isolated at E18.5 and cultivated for 8 (DIV8, top) or 12 days in vitro (DIV12, bottom). *Vti1a^-/-^ Vti1b^+/-^* and *Vti1a^+/ ‑^ Vti1b^-/-^* neurons were stained for GM130 (left), Golgin-97 (middle) and MAP2 (right). Confocal Z-stacks were taken and maximum intensity projections (MIP) were presented. * position of nuclei; arrow heads: Golgi extending into dendrites; Scale bar: middle 10µM, right 20 µM

**
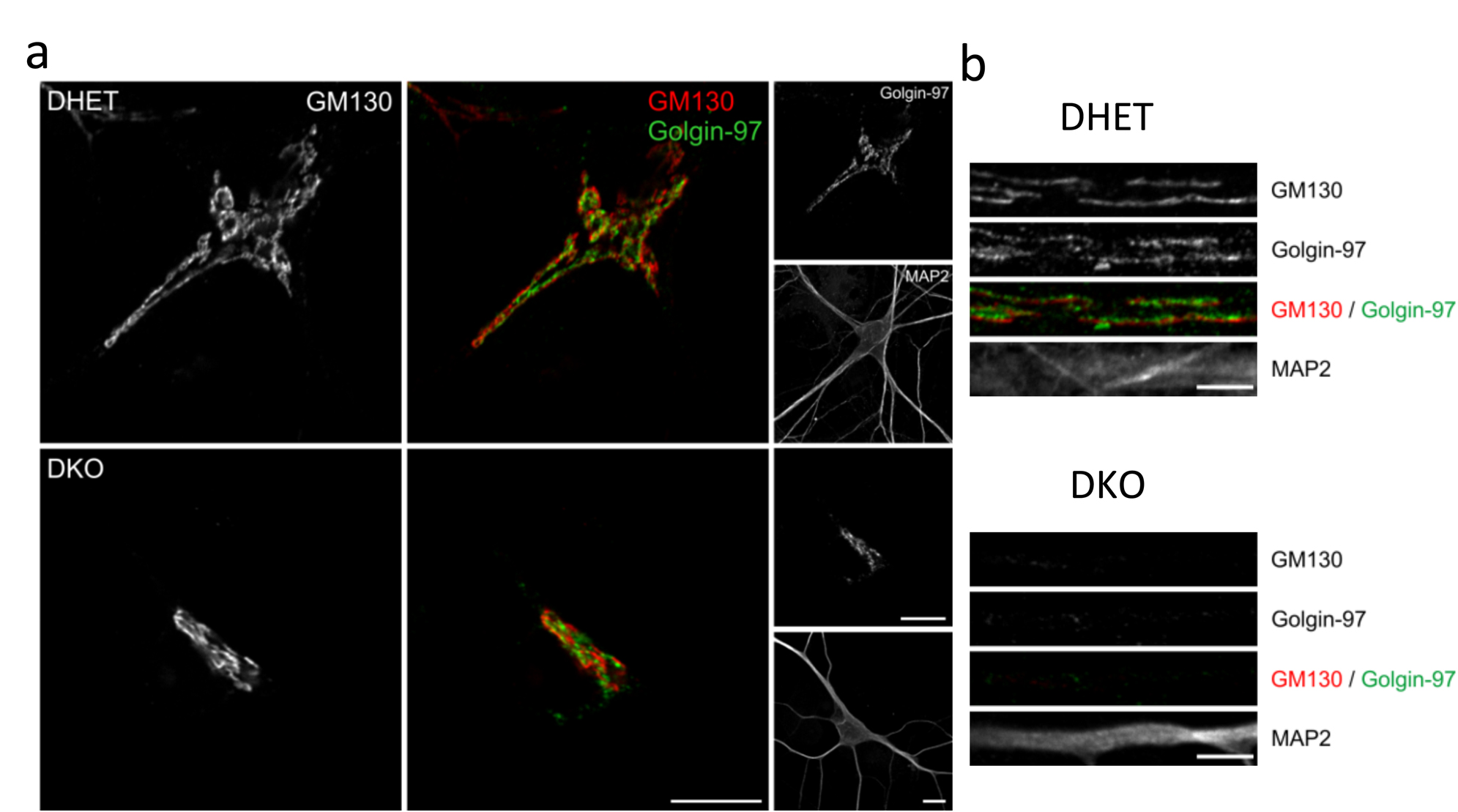
**

**Additional Figure A2: Altered TGN morphology in DKO neurons.** Hippocampal neurons were isolated at E18.5 and cultivated for 8 days in vitro (8 DIV). (a) DHET and DKO neurons were stained for GM130 (white and red), Golgin-97 (green) and MAP2 (white). (b) Dendrites of the cells at DIV12 were magnified, scale bar (a) 10 µm and (b) 5 µm.

**
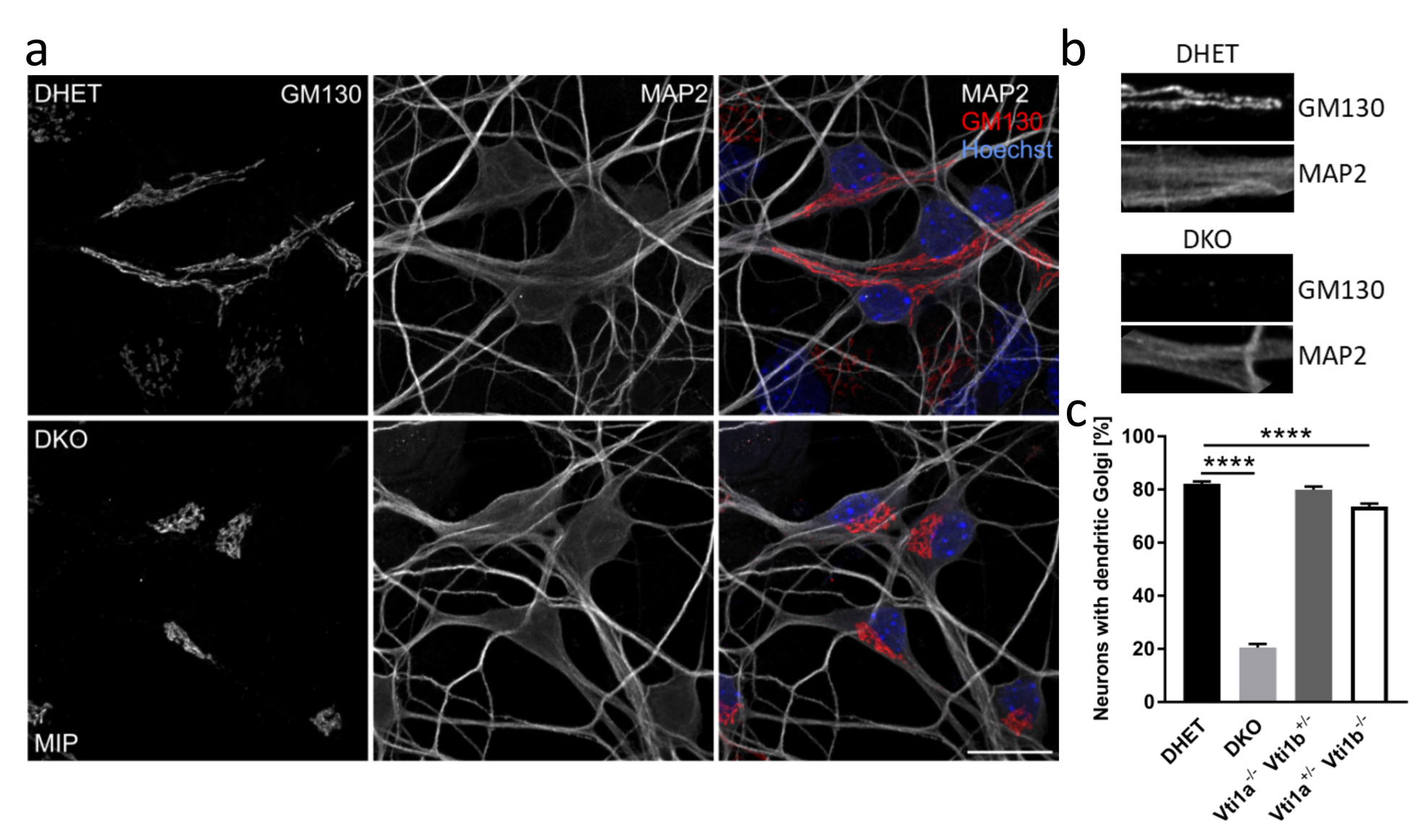
 Additional Figure A3: Less DKO neurons with Golgi extensions at DIV8.** Hippocampal neurons were isolated at E18.5 and cultivated for 8 days in vitro (DIV8). (a) DHET and DKO neurons were stained for GM130 (red) and MAP2 (white). The nuclei were marked with Hoechst (blue). Confocal Z-stacks were taken and maximum intensity projections (MIP) were presented. (b) Enlargements with dendrites in boxed areas. (c) The percentage of cells with Golgi extensions into dendrites was determined for DHET and DKO as well as for *Vti1a^-/-^ Vti1b^+/-^* and *Vti1a^+/ ‑^ Vti1b^-/-^.* N= 3-5 mean of at least 100 cells per experiment. Bars are mean of the mean of 5 different DHET, 4 DKO, 3 *Vti1a^-/-^ Vti1b^+/-^* and 4 *Vti1a^+/ ‑^ Vti1b^-/-^embryos* ± SEM, ****: P<0.0001 one-way ANOVA, scale bar 20 µm

**
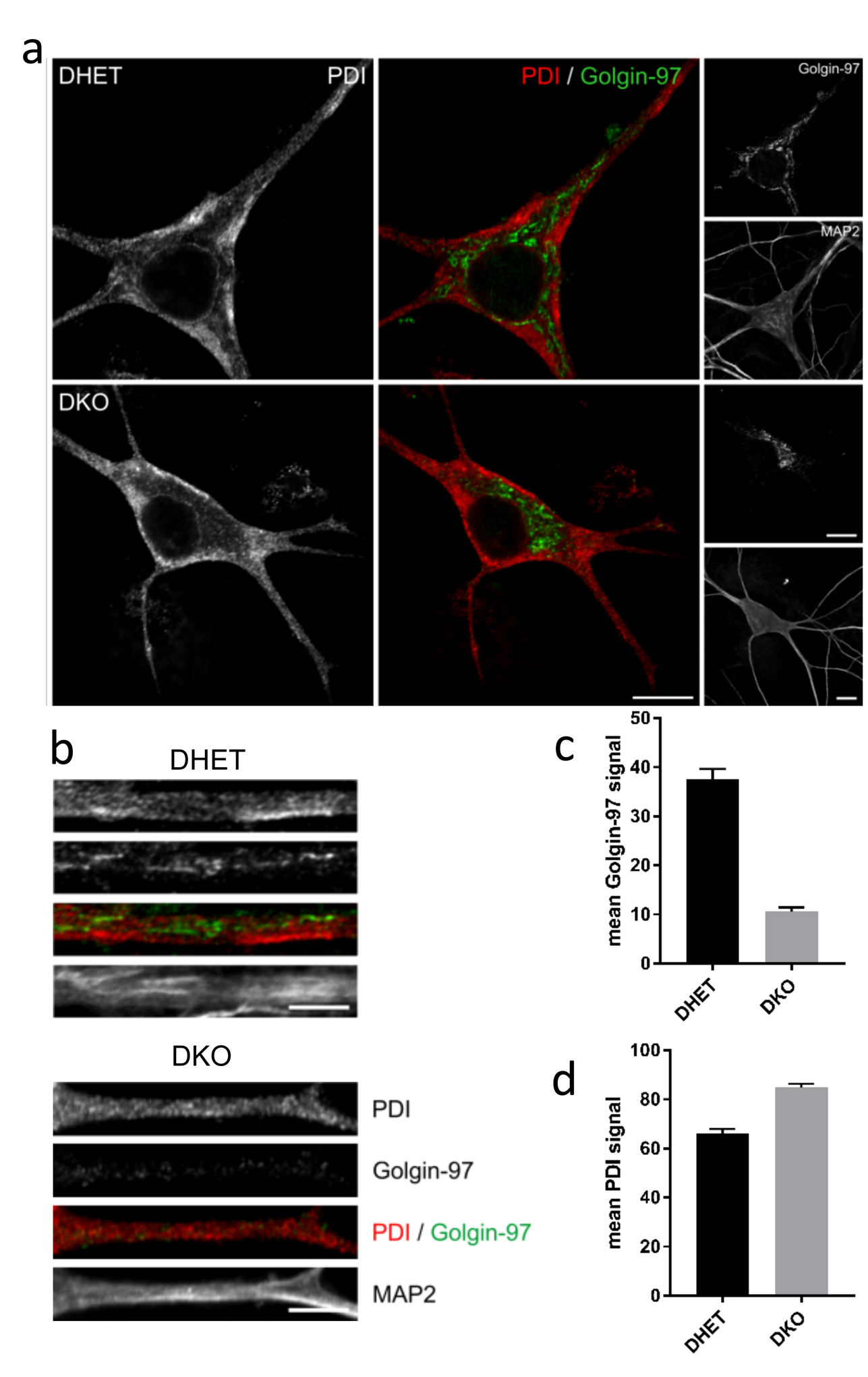
**

**Additional Figure A4: The distribution of ER in DKO appeared to be unaffected.** Hippocampal neurons were isolated at E18.5 and cultivated for 12 days in vitro (12 DIV). (a) DHET and DKO neurons were stained for PDI (white and red), Golgin-97 (green) and MAP2 (white). (b) Dendrites of the cells were magnified. A representative line scan through one dendrite per condition were taken over the length of the dendrite. The mean (c) Golgin-97 and (d) PDI signal intensity of such a line scan was calculated ± SEM, scale bar (a) 10 µm and (b) 5 µm.

**
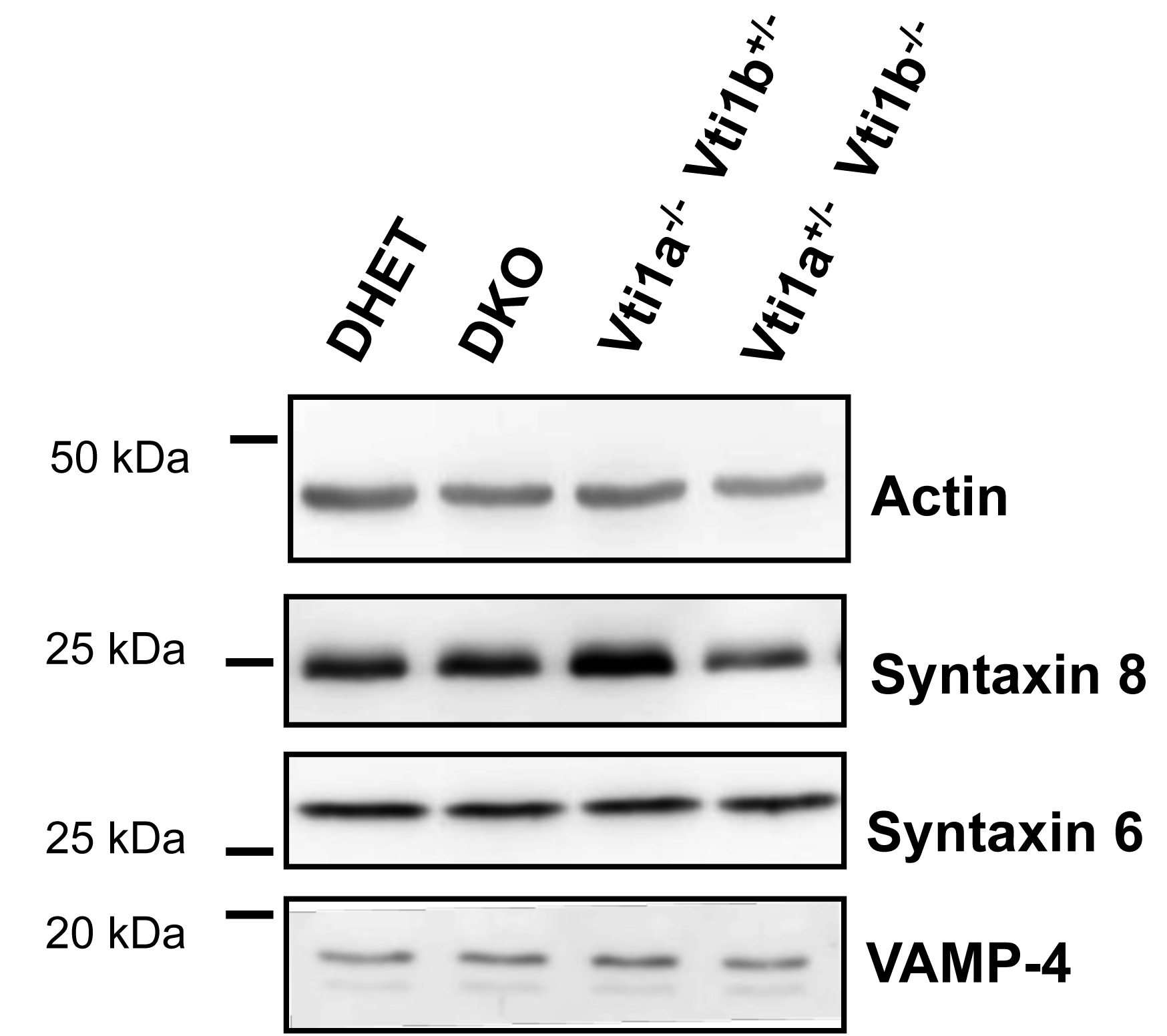
**

**Additional Figure A5: The amounts of three endosomal SNAREs appeared to be unaffected by the absence of vti1a or vti1b in E18.5 brains.** Brain lysates were obtained from different E18.5 embryos of the indicated genotypes. The vti1b SNARE partner syntaxin 8 and the vti1a SNARE partners syntaxin 6 and VAMP-4 were detected in western blots. Representative blots are shown.
